# A Study of Population Size and Activity Patterns and Their Relationship to the Prey Species of the Eurasian Lynx Using a Camera Trapping Approach

**DOI:** 10.3390/ani9110864

**Published:** 2019-10-25

**Authors:** Xiaoming Tang, Shupei Tang, Xiaoyu Li, Dalai Menghe, Wuliji Bao, Changlin Xiang, Fuli Gao, Weidong Bao

**Affiliations:** 1 College of Biological Sciences and Technology, Beijing Forestry University, Beijing 100083, China; tangxm@idsse.ac.cn (X.T.); tangshupei@outlook.com (S.T.); fuligao@bjfu.edu.cn (F.G.); 2 Chifeng Academy of Forestry Sciences, Chifeng 024000, China; yuzi_kuaile@126.com; 3 Inner Mongolia Saihanwula National Nature Reserve Administration, Daban 025150, China; mengdalai2009@163.com (D.M.); 13694759519@139.com (W.B.); xiangchl0612@sina.com (C.X.)

**Keywords:** activity pattern, camera trapping, *Lynx lynx*, meso-carnivores, prey community

## Abstract

**Simple Summary:**

The Eurasian lynx has a wide distribution range in China, but lynx field studies in China are rare compared to those for European populations. Using camera trapping data, this paper reports the lynx population size, activity patterns, and variation within the prey community at a nature reserve in Inner Mongolia. The results found that there were at least 20 lynx in this reserve, and the population has increased steadily over years. There is a seasonal difference in the daily activity rhythms of lynx. The total activity rhythm of lynx is synchronous with five mammal species: hare, red deer, wild boar, raccoon dog, and badger. Red fox and roe deer showed a timed avoidance of lynx to some extent. The present study sets an example for biodiversity conservation; the recovery of prey communities through habitat management should be a priority in determining the restoration of large carnivore populations in China.

**Abstract:**

Revealing the behavioral relationships between predators and their prey is fundamental in understanding the community structure and ecosystem functions of such animals. This study aimed at detecting the population size and activity patterns of Eurasian lynx (*Lynx lynx*) (along with its prey) by camera trapping monitoring from 2014 to 2017 at the Saihanwula nature reserve in central Inner Mongolia. The total effective trapping days were 29,892 and 20 lynx were identified from 343 trapping photos based on the inner side patterns of their forelimbs. The daily activity rhythms of the lynx overlapped with those of different prey in different seasons. The yearly activity pattern of the lynx was influenced by its main prey’s biology. In conclusion, this study reveals that the activity patterns of the top predator matched those of its prey in different time periods. Habitat management strategies promoting the restoration of prey communities would benefit the lynx in maintaining a stable community structure.

## 1. Introduction

Detailed information on population sizes and activity rhythms is crucial in understanding species ecology, adaptation to the environment, and reasonable management strategies for biodiversity conservation. As an important aspect in behavioral ecology, activity pattern studies show animals’ behavioral responses to their environmental factors, including other members in the community [1]. The elusive activity of nocturnal wild cats has made them difficult to study with traditional direct field survey and behavioral techniques [2]. With the quick development of technology such as camera trapping, an effective non-invasive approach, such works are made more feasible for wild cat species [3,4,5]. Several camera-trapping-based field works have been conducted on large wild cat species in China, such as snow leopard (*Panthera uncia*) [6,7,8,9], common leopard (*Panthera pardus*) [8,10], and Amur tiger (*Panthera tigris altaica*) [10,11], which appear in a variety of studies. However, similar camera trapping studies are still needed for other wild cats that urgently require systematic study and protection, including the Eurasian lynx (*Lynx lynx*, called lynx for short).

Eurasian lynx is a wide-spread cat species in Eurasia, including the Scandinavian Peninsula. In Europe and Siberia, field studies on lynx flourished rapidly post World War II, with a number of comprehensive efforts [12,13,14,15,16,17,18,19,20,21]. At present, European Union member states have established a long-term dynamic monitoring system and local area information sharing platform (http://scandlynx.nina.no/) to strengthen conservation. In China, the lynx is listed as a Chinese class II state key protected species and has a large distribution range in China, including in the northeast, northwest, and Tibetan plateau [22,23,24]. Other than a series of studies done by our research group in the Saihanwula national nature reserve, Inner Mongolia [25,26,27,28], only a few sporadic traditional transection surveys on lynx have been carried out in the Xinjiang Uygur Autonomous Region and northeastern area [29,30,31]. Therefore, many types of information are lacking for lynx in China. These characteristics include abundance and activity rhythms. The lynx population in China went through a sharp decline (over two-fold) in size (from 70 thousand to 27 thousand) from the 1970s to 1990s due to over exploitation, poaching, and habitat degradation [22]. For instance, in the 1980s–1990s, the sighting records of live lynx were exceedingly rare (only three records) in the Saihanwula reserve [32]. In the Changbaishan nature reserve, Jilin Province, the density of lynx fell from 0.023/km^2^ in the 1980s to 0.008/km^2^ in the 1990s, and lynx almost disappeared in this area [22]. Thus, the lynx population in China is in danger and it is necessary to establish a long-term monitoring program for lynx populations for purposes of conservation and management. How has the abundance of lynx changed in different habitats over recent years? What is their current status? Currently, these critical questions cannot be answered due to the extreme lack of research on lynx in China. To solve this problem, we require systematic, long term, and species-targeted ecological research programs. A long-term lynx monitoring program has been conducted by our research group at the Saihanwula reserve since 2006, emphasizing population status, restoration, and habitat management. In order to track the abundance trend and status of the lynx in the nature reserve, multiple study approaches were adopted, among which camera trapping has been used since 2009 [25]. There are two objectives of the present paper. Firstly, we seek to update the latest information for the population size and status of lynx in Saihanwula based on camera trapping. Secondly, we desire to study the relationships between lynx and its eight sympatric mammal species through the activity patterns generated from camera trapping. The information provided from our survey will benefit the conservation and management of the lynx in this nature reserve.

## 2. Materials and Methods

### 2.1. Study Area

**Figure 1:**
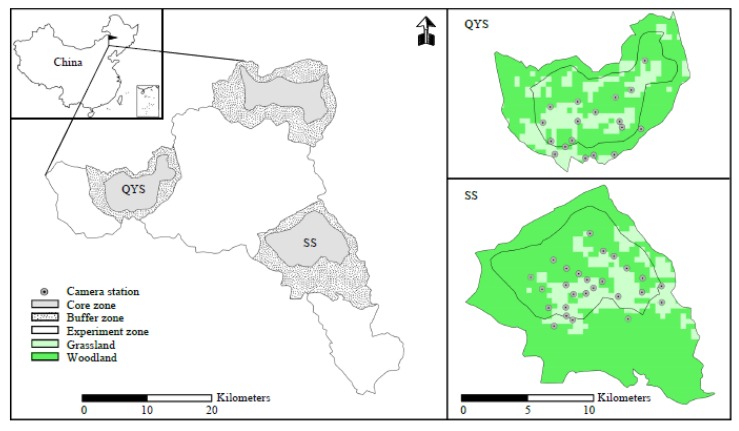
Location of the Saihanwula nature reserve and camera trapping sites.

The Saihanwula national nature reserve is situated in the central part of Inner Mongolia, China (118°18’–118°55’ E, 43°59’–44°27’ N), with an average altitude over 1000 m. It covers about 10.04 × 10^4^ ha of land that consists of forest, scrubland, meadow, and agricultural vegetation. Other than lynx, a diversity of mammal species inhabits Saihanwula. Among them, there are predators, such as wolf (*Canis lupus*), red fox (*Vulpes vulpes*), manul (*Otocolobus manul*), raccoon dog (*Nyctereutes procyonoides*), and badger (*Meles leucurus*). Large- and medium-sized prey includes red deer (*Cervus xanthopygus*), goral (*Naemorhedus griseus*), roe deer (*Capreolus pygargus*), wild boar (*Sus scrofa*), and hare (*Lepus tolai*) [32]. There are three core areas where most human activities (grazing, hunting, farming, and so forth) are forbidden by relevant regulations. Two of these core areas, Shenshan (SS) and Qingyunshan (QYS), were chosen as the sites for this study because of their high frequency of lynx occurrences. The former place occupies an area of 4766 ha and the latter occupies 5745 ha. These two areas are separated by a distance of 20 km and are both covered mainly by woodland and grassland (Figure 1).

### 2.2. Camera Trapping Deployment and Data Analyses

From January 2014 to December 2017, about 50 digital cameras (LTL-5210, Lieke Technology Co. Limited, Shenzhen, China) were positioned in the study area. These monitoring sites were decided mainly based on previous snow tracking, transection surveys, and camera studies. That is, the cameras were mainly placed around positions where lynx had been pictured or left their snowy footprints and feces. After these positions were set, the remainder of the camera was placed around sites where ungulates appeared frequently. The distance between any two camera sites was greater than 1 km. The cameras were also moved when the animals were barely pictured at their original sites or there were new places with traces of lynx. In total, there were 44 different sites, which covered most of the area of SS (26 cameras) and the southern part of QYS (18 cameras) (Figure 1). The inner forelimb markings of the lynx were used for individual identification in this study (examples are shown in Figure S1). For this purpose, cameras were attached perpendicular to the animal route and at around 30 cm above the ground, and slight adjustments were made in accordance with the vegetation growth conditions in different seasons. The photographical pattern was set to take one photo, followed by a 15 s video with an interval of 1 min and was left working all day. Our lynx did not show any fear from the infrared lights and the operators’ odor left on the digital cameras; some individuals even marked their visits by urinating on the camera fixing trees. The cameras were checked once a month, and the SD cards and batteries were replaced.

When checking the photos and videos, once an animal appeared, detailed information was recorded, including the location, date, time, numbers in the group, and behaviors.

For the activity rhythm, each occurrence (photos and videos) is regarded as one single independent capture (IC). The relative IC (RIC) was used to compare the yearly and daily activity patterns. The formulas of the RIC are as follows:

(1) $$\mathrm{yearly}\;\mathrm{RIC}=\;\frac{\mathrm{IC}\;\mathrm{of}\;\mathrm{particular}\;\mathrm{year}}{\mathrm{camera}\;\mathrm{trap}\;\mathrm{day}\;\mathrm{of}\;\mathrm{particualr}\;\mathrm{year}}\;\times 1000$$

(2) $$\mathrm{monthly}\;\mathrm{RIC}\;\mathrm{of}\;\mathrm{other}\;\mathrm{animals}=\;\frac{\mathrm{IC}\;\mathrm{of}\;\mathrm{particular}\;\mathrm{month}}{\mathrm{camera}\;\mathrm{trap}\;\mathrm{day}\;\mathrm{of}\;\mathrm{particualr}\;\mathrm{month}}\;\times 100$$

(3) $$\mathrm{monthly}\;\mathrm{RIC}\;\mathrm{of}\;\mathrm{lynx}=\;\frac{\mathrm{IC}\;\mathrm{of}\;\mathrm{particular}\;\mathrm{month}}{\mathrm{camera}\;\mathrm{trap}\;\mathrm{day}\;\mathrm{of}\;\mathrm{particular}\;\mathrm{month}}\;\times \;1000$$

(4) $$\mathrm{daily}\;\mathrm{RIC}=\;\frac{\mathrm{IC}\;\mathrm{of}\;\mathrm{particular}\;\mathrm{time}\;\mathrm{phase}\;}{\mathrm{total}\;\mathrm{IC}}\;\times \;100$$

For the daily activity rhythm, the time phase was one hour. For example, 0:00–0:59 was time phase No. 0, and 1:00–1:59 was time phase No. 1. To compare the daily activity rhythms in different seasons, the study years were divided into four seasons: spring (March–May), summer (June–August), autumn (September–October), and winter (November–February next year). Fridenman’s test was applied for a comparison amongst the different seasonal daily activity rhythms of the lynx. While for the activity rhythms of the lynx and its prey species, Pearson correlation tests were used, with *p* < 0.05 as the significance level.

## 3. Results

### 3.1. Population Abundance

A sampling effort of 29,892 camera-days with 343 independent capture cases of lynx was obtained from 2014 to 2017 (Table 1). The total RIC was 11.47, and there were 10 lynx groups during the four study years. At least 20 lynx had lived in the whole study area with a density of 27.14/100 km^2^. In 2014, five lynx groups were pictured, including a mother–kitten pair that had been photographed twice, a female individual taking the sub-adults, and two sibling groups, both featuring two individuals. However, in 2015, the RIC decreased acutely to 8.02, and no groups were found. In 2016, the RIC recovered, and one sibling group was recorded. For 2017, the RIC of 14.06 reached its highest point during the survey years.

According to the videos taken in this work, the lynx did not show any fear of the infrared light or the scent of the camera operators. Indeed, the lynx approached the cameras and sometimes even left urine marks around them. Thus, the camera trapping technique is suitable for monitoring lynx activity.

### 3.2. Distribution of Lynx

**Figure 2:**
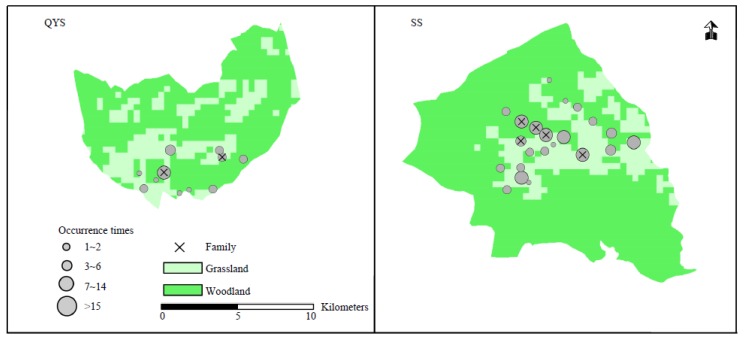
Distribution of independent captures and family sites for the lynx in Qingyunshan (QYS) and Shengshan (SS).

The position of lynx family groups showed that the distribution was quite dispersive, with only one site that had over 15 records and two family sites found in QYS (Figure 2). In comparison, the family sites were more focused, with high frequency sites in the middle part of SS.

### 3.3. Activity Rhythm of Lynx

#### 3.3.1. Monthly Activity Rhythm

**Figure 3:**
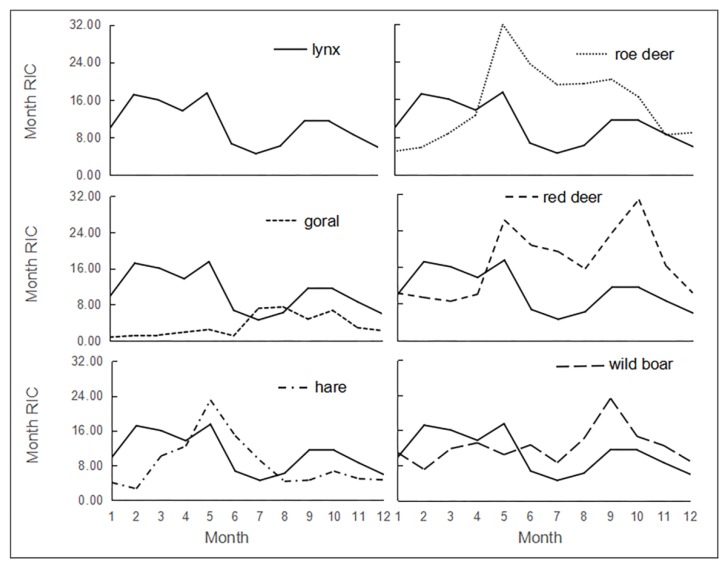
The monthly activity pattern of lynx and its main prey in Saihanwula.

Lynx presented a monthly bimodal pattern, with two peak periods overlapping with the herbivores’ active peaks to a certain degree (Figure 3). The main peak of lynx started in February and ended in May, which was also the peak month for roe deer, red deer, and hare. The second peak of the lynx’s yearly active pattern appeared in September and October, with the September peak overlapping with the wild boar and the October peak overlapping with the red deer. The less active months for the lynx in Saihanwula were June, July, August, and December. When it came to December, all species showed low activity.

#### 3.3.2. Daily Activity Rhythm

**Figure 4:**
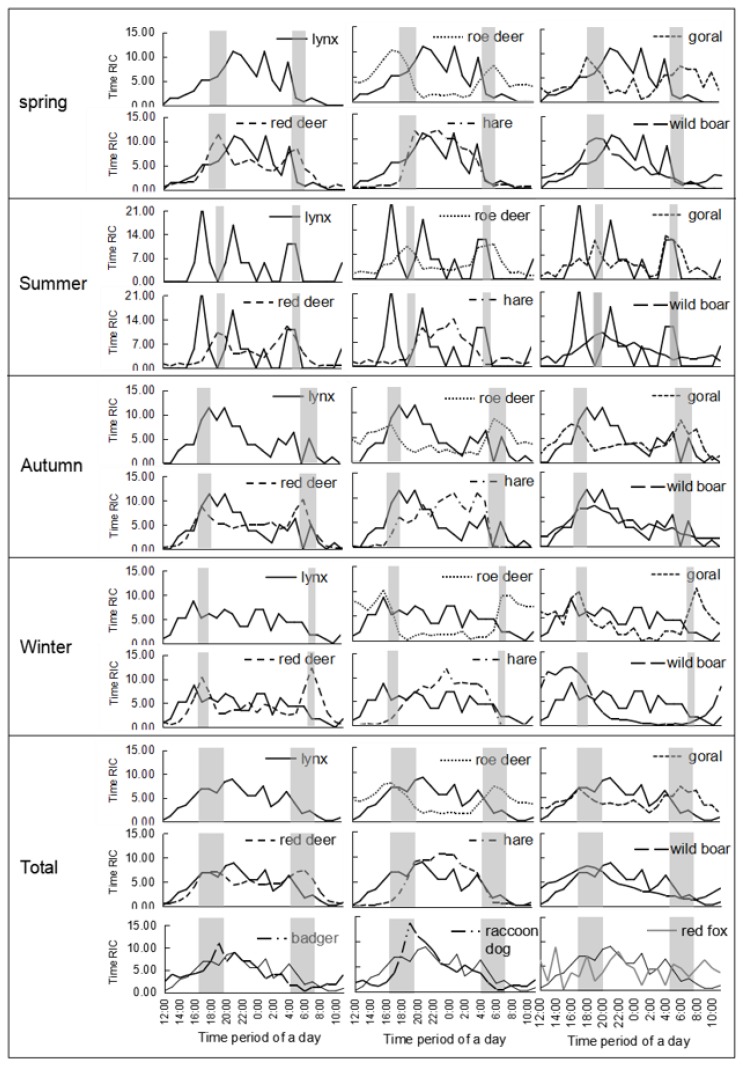
Daily activity rhythm of the lynx and sympatric prey in different seasons (grey bars show sunset and sunrise; data from https://richurimo.51240.com).

The daily activity rhythm of the lynx generally presented multiple peaks, with more active hours at night (as well as twilight) and inactive hours at midday in our study area. Although the Frideman’s test showed that there were no significant differences among seasonal daily activity rhythms (*p* = 0.141 > 0.05), some visible variations were found (Figure 4). The spring’s active rhythm was the most nocturnal while the autumn rhythm was the most crepuscular. The active rhythms in the summer and winter were the transitional forms between them. Amongst all four seasons, lynx was the most nocturnal in spring, with all active peaks at night and the main peak at 21:00–22:00, the second one at 1:00–2:00, and the third one at 4:00–5:00. When it came to summer, the main peak moved forward to 16:00–17:00, with the second peak at 21:00–22:00, and the last peak at 4:00–6:00, which indicates that the rhythm distribution became more crepuscular. This variation tendency remained for the autumn pattern. The autumn pattern possessed the most active peak, lasting from 17:00 to 20:00, and an active period right before sunrise from 3:00 to 5:00. When winter arrived, the frequency of lynx activity increased in the afternoon and at night, with an unapparent peak before sunset at 16:00–17:00 (Figure 4). In general, the four seasons had four activity peaks at 1:00–2:00, 4:00–5:00, 17:00–19:00, and 20:00–22:00 (by time order).

Red deer (*r* = 0.644, *p* = 0.001 < 0.01), hare (*r* = 0.712, *p* = 0.000 < 0.01), wild boar (*r* = 0.563, *p* = 0.004 < 0.01), badger (*r* = 0.669, *p* = 0.000 < 0.01), and raccoon dog (*r* = 0.730, *p* = 0.000 < 0.01) were extremely synchronized with the lynx’s total daily activity rhythm (Table 2). While the roe deer (*r* = −0.220) and red fox (*r* = −0.059) showed a timed avoidance to lynx to some extent, the relationship between the lynx and other sympatric prey species varied in different seasons. In spring, there were highly positive correlations between three species and the lynx, based on a Pearson correlation test: hare (*r* = 0.847, *p* = 0.000 < 0.01), red deer (*r* = 0.588, *p* = 0.002 < 0.01), and wild boar (*r* = 0.620, *p* = 0.001 < 0.01), of which hare was highly synchronized with the lynx in its unimodal and nocturnal daily activity pattern. Although the three ungulates presented similar crepuscular activity patterns in spring, unlike red deer, roe deer (*r* = −0.311) and goral (*r* = −0.229) showed an active avoidance of lynx to some extent. In spite of the activity rhythm synchronization between ungulates and lynx during the sunrise period, the summer activity rhythms showed no significant relationships between the lynx and its five main types of prey. In autumn, the activity pattern of the lynx was positively related to that of red deer (*r* = 0.507, *p* = 0.011 < 0.05), wild boar (*r* = 0.856, *p* = 0.000 < 0.01), and roe deer (*r* = 0.507, *p* = 0.011 < 0.05); the two deer species and the lynx were crepuscular, with peaks at sunrise and sunset. The negative correlation between the daily winter activity patterns of lynx and roe deer (*r* = −0.421, *p* = 0.041 < 0.05) was remarkable, but the activity of the hare was positively correlated to that of the lynx (*r* = 0.460, *p* = 0.024 < 0.05).

## 4. Discussion

The onset of lynx population recovery in our study area occurred after the establishment of the reserve and the declaration of the relevant regulations on illegal hunting in 1997. Before that, there were only three records of lynx, two in 1987 and one in 1996 [32]. Our research group has been tracking local lynx population dynamics continuously since 2006 [25,27]. In 2006–2009, a snowy footprint tracking survey indicated that there were over seven individuals in the SS and QYS areas, which implied a minimum density of 6.66/100 km^2^. During 2010–2012, this number was confirmed through camera trapping in the SS area, with a minimum density of 12.18/ 100 km^2^. Combined with this study, a conclusion could be made that the lynx population in the Saihanwula nature reserve has been increasing since 2006. The density of the lynx was 1.32 ± 0.151/100 km^2^ [30] in the northeast area of China, 1.00 ± 0.70/100 km^2^ in the Changbaishan nature reserve, and 0.50/100 km^2^ in Xinjiang [29]. In comparison, the lynx has a considerably higher density in the Saihanwula nature reserve. In general, judging by population density along with breeding situation, the lynx population in the Saihanwula reserve has undergone restoration and reached an optimistically high population size. The reason for the low RIC and lack of a lynx family recorded in 2015 is because an illegal grazing case occurred in the reserve, and some of the cameras were destroyed by the herders.

The increase in the lynx population is possibly attributable to the recovery of ungulate prey after a feeding program initiated in 2009, where the nature reserve staff periodically offered nutrient mineral blocks to herbivores in the reserve. This forage implementation may provide informative empirical data for nature reserves aiming at large carnivore conservation through the restoration of prior prey.

The daily activity rhythms of wild cat species are usually nocturnal or crepuscular, such as with puma (*Panthera onca*), jaguar (*Puma concolor*), Iriomote cat (*Prionailurus iriomotensis*), and leopard cat (*Prionailurus bengalensis*) [1,33,34]. Species in the Lynx genus also accord with this conclusion, including the Eurasian lynx [35,36], Canadian lynx (*Lynx canadensis*) [37,38], and Iberian lynx (*Lynx pardinus*) [39]. The activity rhythms of carnivores are affected by a number of factors, including the moon phase, prey rhythms, inter-specific competition, gender, and reproductive phases [40,41,42]. Among these factors, prey abundance plays the most important role [1]. The active bouts of hunting during a predator’s day usually synchronize with the schedules of their main prey to raise their hunting success rate [43,44]. The main prey species of lynx are hare and ungulates [17,18,19,45,46,47,48,49] with nocturnal or crepuscular activity rhythms, which explains why the lynx’s daily activity patterns are nocturnal or crepuscular. For example, in north Scandinavia, lynx in different latitudinal areas adjusted their activity rhythms to synchronize with the main local ungulate species [35]. Through an analysis of 35 lynx feces samples in Saihanwula, the hare was found to be the most important prey, with 29 detections [26]; the hare’s activity pattern was synchronized with lynx at the highest level (Table 2), followed by the roe deer, with four detections [26]. However, the activity rhythm of roe deer had no synchronization and even showed an avoidance of lynx based on this study (Figure 4 and Table 2). The reason for this result is possibly the high population abundance of roe deer, with a total yearly RIC of 129.16, which was over 11 times of that lynx. The abundance was so high that it was unnecessary for lynx to synchronize their activity rhythms with roe deer to increase hunting success. Both red deer and wild boar had only one detection in the fecal analysis [26], which indicated that they were not the main prey but more occasional choices for lynx under circumstance of insufficient hare or roe deer. Compared with hare and roe deer, red deer and wild boar are less suitable prey with a large size and aggressiveness. Thus, there was no need for them to avoid lynx, and their active periods highly overlapped with those of the lynx.

There was obvious seasonal variation in the activity rhythm of the lynx [36,37], and this study showed the same result (Figure 4). This variation may relate to the prey, thermoregulation, sex, or breeding status [36,37]. Some reports show that lynx changed their main prey during different seasons. In the warm seasons, lynx prefer small prey, while in cold seasons, they mainly prey on larger animals [50,51]. The fecal analyses in Saihanwula agreed with this conclusion [26]. Thus, in the most active period of hare during spring in Saihanwula (Figure 3), the lynx exhibited a totally nocturnal pattern to synchronize with the hare’s activity rhythm (Figure 4 and Table 2). In autumn, the lynx became more crepuscular to synchronize with the roe deer (Figure 4 and Table 2), which showed a relatively high activity pattern (Figure 3). Environmental temperature may play a partial role in the seasonal differences of the lynx’s activity rhythms. Studies on Canadian lynx and Iberian lynx revealed a pronounced increase of diurnal activity in winter [37,38,52], but this time budget shift has not yet been reported for Eurasian lynx [36,53]. Our study provides solitary proof that Eurasian lynx evidently increase their activity in the afternoon during winter (Figure 4), which may result in lower energy expenditure under cold temperatures.

The co-existence of top-predators and meso-predators is always related to niche segregation [39]. Top predators might suppress the abundance of meso-predators by killing them [54]. Red fox are often reported to be killed by lynx in different areas [55,56]. The red fox preys mainly on rodent and hare and has a highly overlapping food niche with the lynx [57], which may indicate a food competition with the lynx and a risk of being killed by the lynx. To co-exist with lynx, one of its strategies is to compromise its activity times [58]. Our results may also indicate a similar situation—that the abundance of red fox in our study area was low, with an independent capture number of 115; and a negative correlation in daily activity pattern (Table 2). The badger and raccoon dog in Saihanwula did not show any activity avoidance of the lynx. The badger’s main prey are insects and plants in Saihanwula [59], which means its food niche does not overlap with that of the lynx. Thus, they do not need to occupy a different time niche from the lynx to co-exist with the lynx. Another hypothesis is that the badger is just indifferent and shows no avoidance of the lynx, even with the same main prey in Spain [58]. The raccoon dog preys mainly on ground nesting bird and rodent species, which overlaps with the lynx to a small degree. However, its activity rhythm preset shows no avoidance of the lynx, so there might be other ways for it to escape the lynx’s predation.

There are some drawbacks to this study. With the limited cameras we were not able to cover the whole reserve. Thus, there might be lynx individuals that have not been pictured. Moreover, since the lynx was our study priority, there is the possibility that impacts from other important carnivore species, such as the wolf, have been underestimated or ignored. Other than that, the camera trapping data for the summer were insufficient, as we had to remove some of our cameras during this time to prevent the cameras from being destroyed or stolen by herders or poachers. Current data on the photo identification of the lynx in Saihanwula is not enough for further research, such as population size estimation through the capture–recapture model and home range calculation. We hope to conduct studies at a deeper level based on photo IDs. For the predator–prey relationship in Saihanwula, the next step is to study spatial interactions to construct a spatial–temporal network of mammal communities, which will enable us to have a more comprehensive view of the ecosystem where the lynx lives.

## 5. Conclusions

The present study found that the Eurasian lynx population is recovering. We speculate that the restoration of prey abundance is the basic causation, although it is not possible to demonstrate this from the presently available data. The activity pattern and daily rhythm of the lynx followed the pace of different prey resources seasonally. Red foxes avoided direct conflict with, and predation risk from, the lynx by adjusting its activity pulse and maintaining its co-existence with the top predator. The camera trapping technique is suitable to determine population trends and the behavioral monitoring of lynx because our animals used the camera fixing trees as convenient scent marking sites. However, we still could not estimate the exact population of the lynx in the study region with the present method of camera trapping because our sample lynx lacked any significant body patterns. We hope to tackle this problem with non-invasive sampling DNA identification, which may provide some insights into the population structures and kinship relations of lynx.

## Figures and Tables

**Table 1:** Yearly relative independent capture and abundance of lynx based on camera trapping.

Year	Trap Days	IC	Yearly RIC	Min. Numbers in SS	Min. Numbers in QYS	No. of Groups
2014	8357	94	11.25	≥5	≥7	5
2015	7606	61	8.02	≥7	≥1	0
2016	5395	68	12.60	≥5	≥3	1
2017	8534	120	14.06	≥5	≥6	4
Total	29,892	343	11.47	≥10	≥10	10

IC: Independent capture; RIC: Relative independent capture; SS: Shenshan; QYS: Qingyunshan.

**Table 2:** Pearson correlation test between the lynx and its sympatric prey.

Seasons		Roe Deer	Goral	Red Deer	Hare	Wild Boar	Badger	Raccoon Dog	Red Fox
Spring	*r*	−0.311	−0.229	0.588 **	0.847 **	0.620 **			
	*p*	0.139	0.281	0.002	0.000	0.001			
Summer	*r*	0.212	0.383	0.212	0.135	0.256			
	*p*	0.321	0.065	0.321	0.528	0.227			
Autumn	*r*	0.507 *	0.109	0.507 *	0.400	0.856 **			
	*p*	0.011	0.611	0.011	0.053	0.000			
Winter	*r*	−0.421 *	−0.180	0.132	0.460 *	0.098			
	*p*	0.041	0.400	0.537	0.024	0.648			
Total	*r*	−0.220	0.128	0.644 **	0.712 **	0.563 **	0.669 **	0.730 **	−0.059
	*p*	0.302	0.552	0.001	0.000	0.004	0.000	0.000	0.784

* *p* < 0.01, ** *p* < 0.05.

## Supplementary Materials

The following are available online at https://www.mdpi.com/2076-2615/9/11/864/s1, Figure S1: Six photos showing the inner side of forelimbs. (**a**) Two black dots on left forelimb; (**b**) One bigger black dot on left forelimb; (**c**) No obvious dot on right forelimb; (**d**) Three heavy black dots on left forelimb; (**e**) “V” type black mark on left forelimb; (**f**) Three light black dots on left forelimb.
