# Supplementary material for: A Study of Population Size and Activity Patterns and Their Relationship to the Prey Species of the Eurasian Lynx Using a Camera Trapping Approach
Source: Animals (Basel). 2019 Oct 25;9(11):864. doi: 10.3390/ani9110864 (PMC6912215; doi:10.3390/ani9110864)
Supplement: Supplementary file 1 [file animals-09-00864-s001.pdf]

animals

MDPI

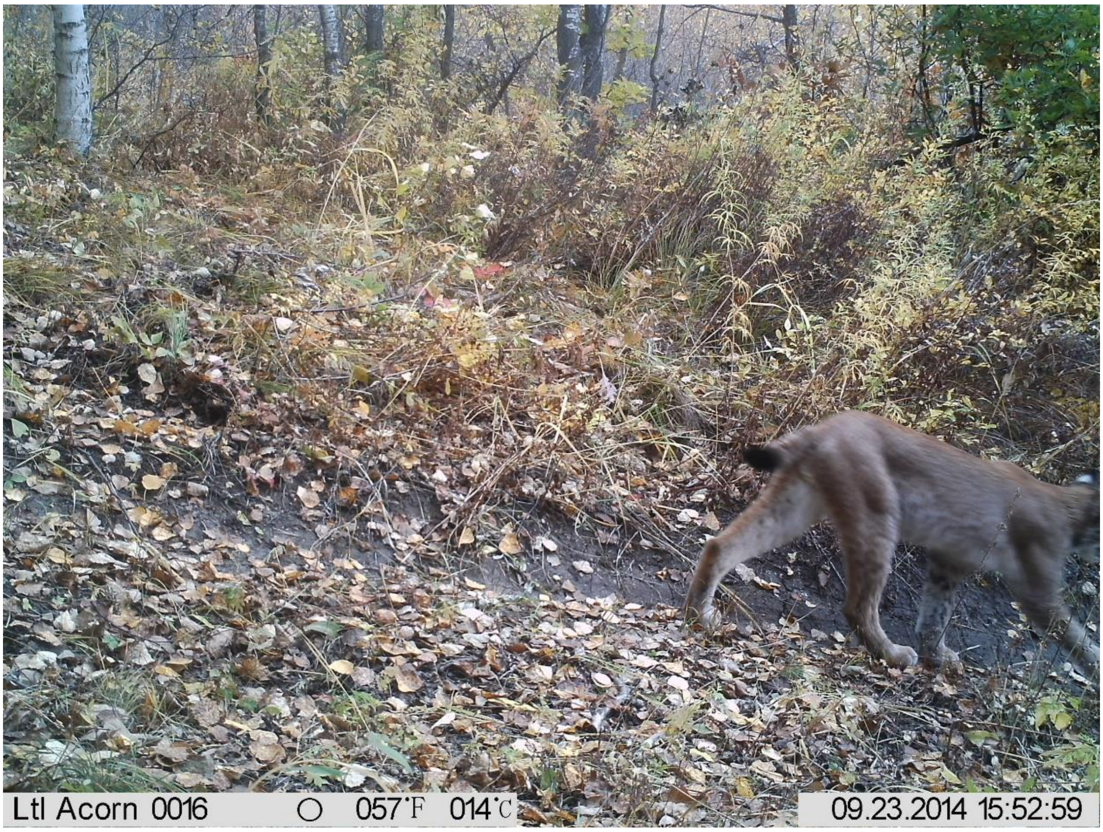

(a)

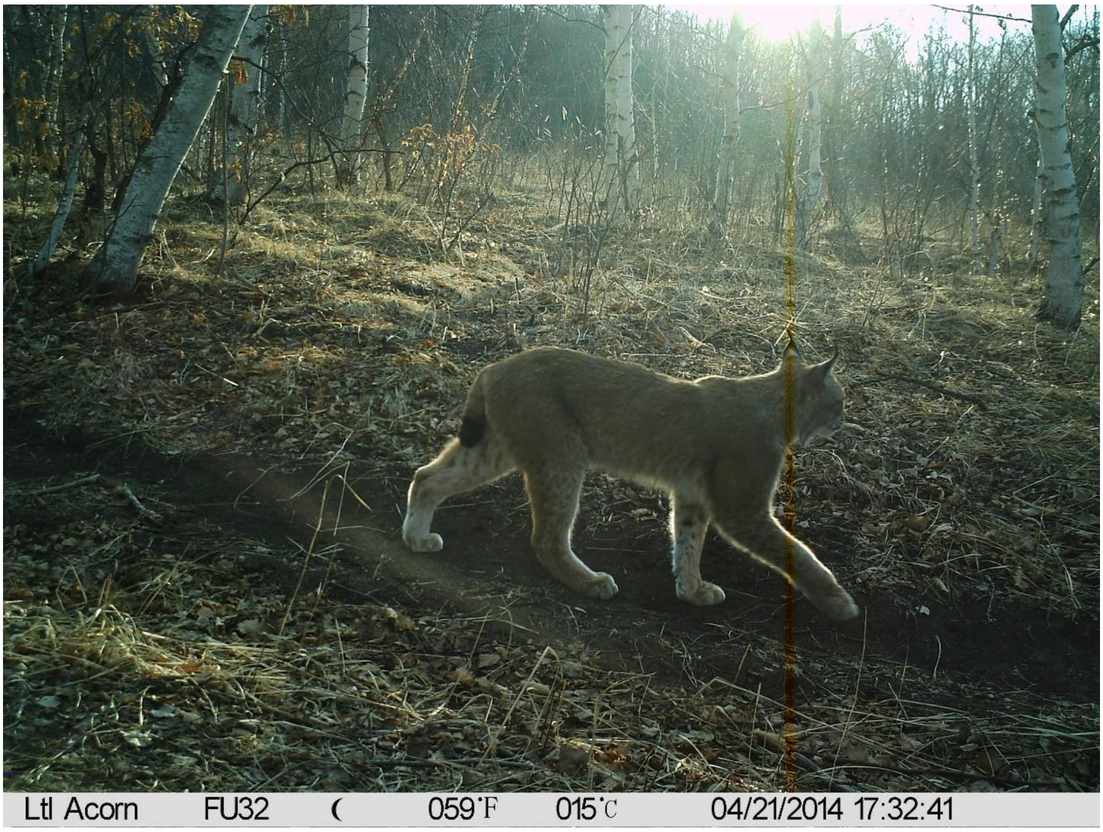

(b)

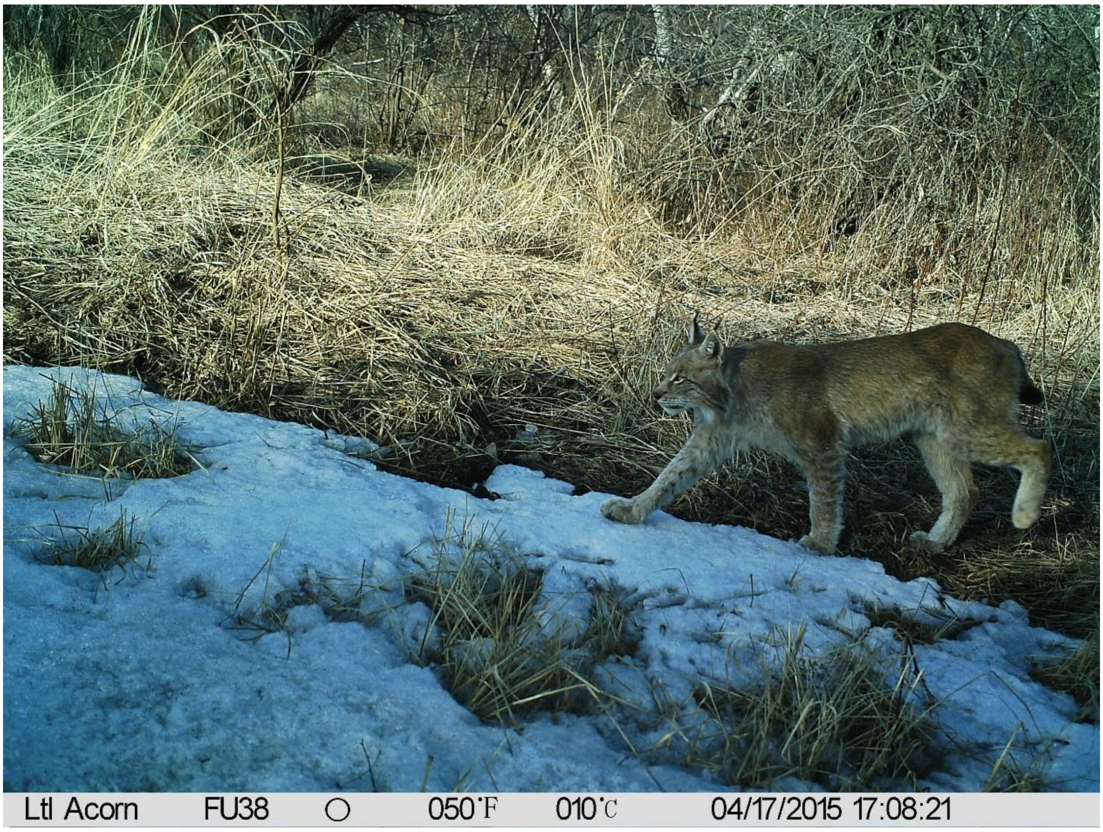
Ltl Acorn FU38 050°F 010°C 04/17/2015 17:08:21

(c)

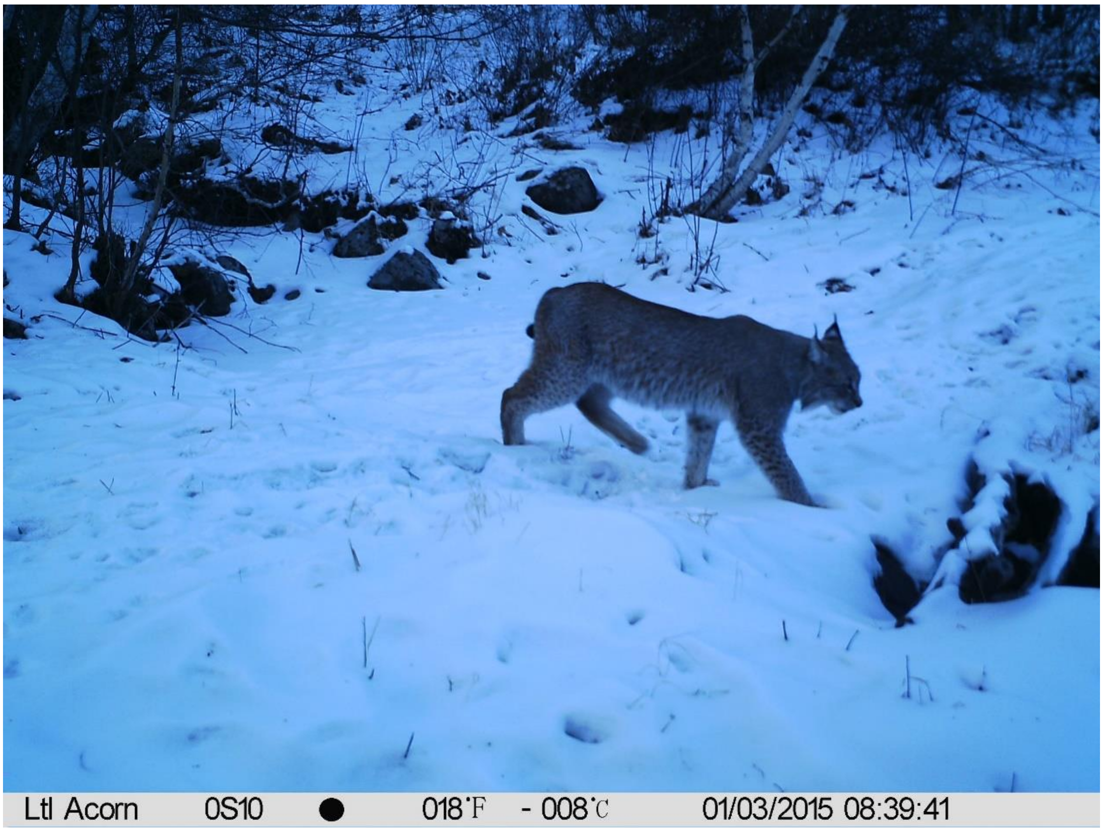
Ltl Acorn 0S10 018°F - 008°C 01/03/2015 08:39:41

(d)

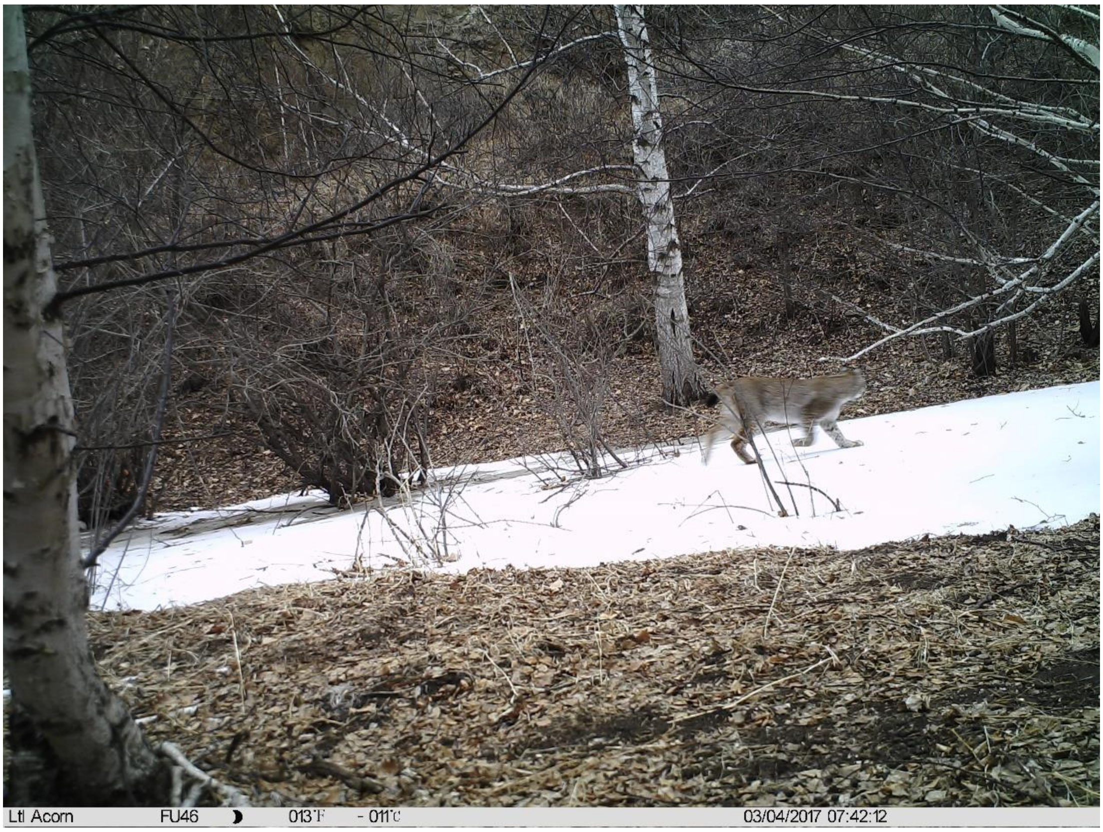

(e)

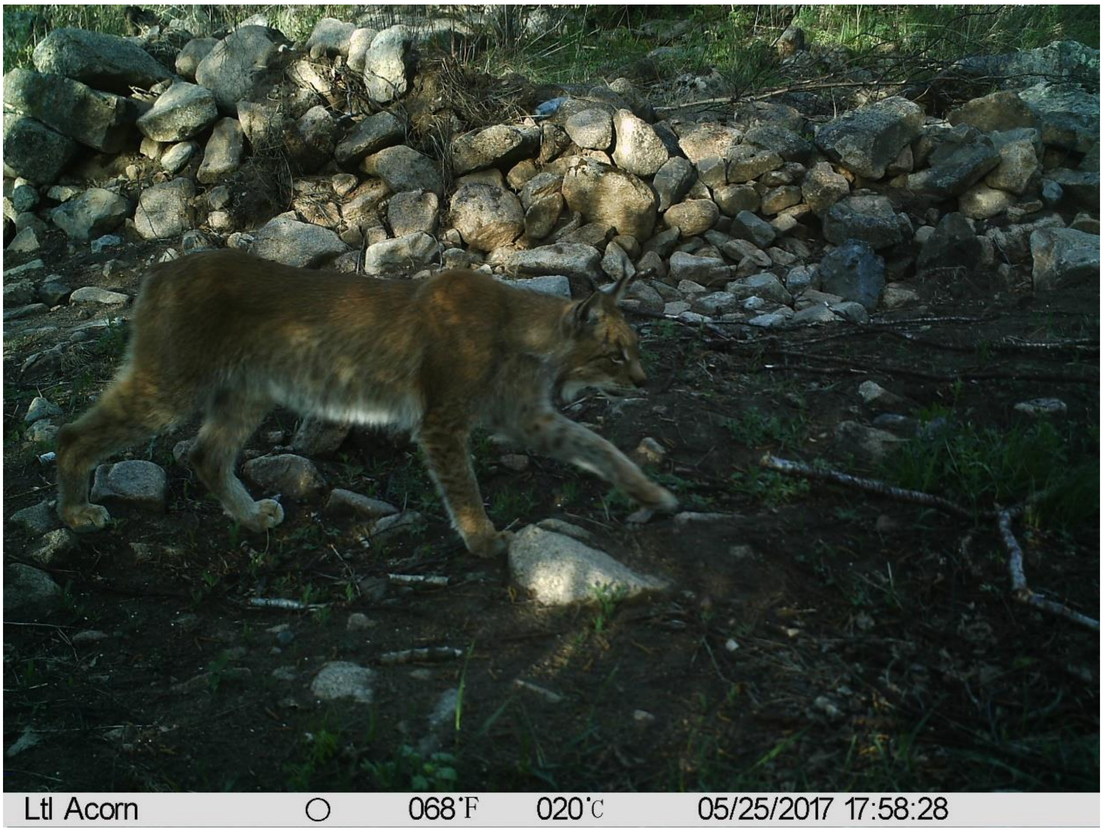

(f)

Figure S1. Six photos showing the inner side of forelimbs. (**a**) Two black dots on left forelimb; (**b**) One bigger black dot on left forelimb; (**c**) No obvious dot on right forelimb; (**d**) Three heavy black dots on left forelimb; (**e**) "V" type black mark on left forelimb; (**f**) Three light black dots on left forelimb.
